# (2,2′-Bipyridine-6,6′-dicarboxyl­ato-κ^3^ *N*,*N*′,*O* ^6^)(6′-carb­oxy-2,2′-bipyridine-6-carboxyl­ato-κ^3^ *N*,*N*′,*O* ^6^)rhodium(III)

**DOI:** 10.1107/S1600536812004850

**Published:** 2012-02-17

**Authors:** Huimin Wang, Xiaojun Gu, Bingbing Zhang, Haiquan Su, Ming Hu

**Affiliations:** a College of Life Sciences, Inner Mongolia University, Hohhot 010021, People’s Republic of China; b School of Chemistry and Chemical Engineering, Inner Mongolia University, Hohhot 010021, People’s Republic of China

## Abstract

The Rh^III^ ion in the title compound, [Rh(C_12_H_6_N_2_O_4_)(C_12_H_7_N_2_O_4_)], is coordinated by four N atoms and two O atoms from two chelating ligands *L* and *HL* (*H_2_L* = 2,2′-bipyridine-6,6′-dicarb­oxy­lic acid) to form a distorted octa­hedral geometry. Face-to-face π-stacking inter­actions are observed between inversion-related pyridine rings, with a centroid-to-centroid distance of 3.581 (1) Å [the perpendicular distance between the rings is 3.3980 (7) Å]. Inter­molecular O—H⋯O hydrogen bonds link adjacent mol­ecules into one-dimensional supra­molecular chains along the *c* axis, while several inter­molecular C—H⋯O inter­actions are also observed.

## Related literature

For structures and photophysical properties of *Ln* ^III^ (*Ln* is a lanthanide) complexes with the title ligand, see: Bünzli *et al.* (2000 ▶). For Rh complexes with pyridyl triazole ligands, see: Burke *et al.* (2004 ▶). For an Mn–Rh coordination polymer with the 2-methyl­pyrazine-5-carb­oxy­lic acid ligand, see: Chapman *et al.* (2002 ▶). For a *catena*-poly diaqua Cd^II^ complex with the title ligand, see: Knight *et al.* (2006 ▶). For a review reporting the properties of coordination polymer networks *via* O and N atoms, see: Robin & Fromm (2006 ▶). For the structures and thermal properties of five *Ln* complexes with the title ligand, see: Wang *et al.* (2010 ▶). For a related Ni^II^ complex with the title ligand, see: Wang *et al.* (2009 ▶).
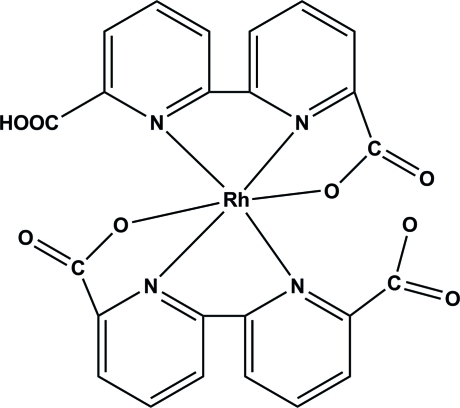


## Experimental

### 

#### Crystal data

[Rh(C_12_H_6_N_2_O_4_)(C_12_H_7_N_2_O_4_)]
*M* *_r_* = 588.29
Monoclinic, 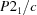

*a* = 9.3308 (4) Å
*b* = 13.6186 (6) Å
*c* = 16.9974 (8) Å
β = 100.696 (1)°
*V* = 2122.37 (16) Å^3^
*Z* = 4
Mo *K*α radiation
μ = 0.87 mm^−1^
*T* = 296 K
0.2 × 0.2 × 0.2 mm

#### Data collection

Bruker APEXII CCD area-detector diffractometer
15424 measured reflections
5269 independent reflections
4714 reflections with *I* > 2σ(*I*)
*R*_int_ = 0.017

#### Refinement

*R*[*F* ^2^ > 2σ(*F* ^2^)] = 0.023
*wR*(*F* ^2^) = 0.061
*S* = 1.03
5269 reflections
335 parameters
H-atom parameters constrained
Δρ_max_ = 0.42 e Å^−3^
Δρ_min_ = −0.58 e Å^−3^

### 

Data collection: *APEX2* (Bruker, 2008 ▶); cell refinement: *SAINT* (Bruker, 2008 ▶); data reduction: *SAINT*; program(s) used to solve structure: *SHELXS97* (Sheldrick, 2008 ▶); program(s) used to refine structure: *SHELXL97* (Sheldrick, 2008 ▶); molecular graphics: *DIAMOND* (Brandenburg & Putz, 2006 ▶); software used to prepare material for publication: *publCIF* (Westrip, 2010 ▶).

## Supplementary Material

Crystal structure: contains datablock(s) I, global. DOI: 10.1107/S1600536812004850/zq2151sup1.cif

Structure factors: contains datablock(s) I. DOI: 10.1107/S1600536812004850/zq2151Isup2.hkl

Additional supplementary materials:  crystallographic information; 3D view; checkCIF report

## Figures and Tables

**Table:** 

Rh1—N1	1.9529 (13)
Rh1—N4	1.9571 (14)
Rh1—O1	2.0226 (12)
Rh1—O5	2.0316 (13)
Rh1—N3	2.0689 (15)
Rh1—N2	2.0808 (14)

**Table:** 

N2—C6—C5—N1	−2.1 (2)
N4—C15—C16—N3	−1.8 (2)

**Table 2:** Hydrogen-bond geometry (Å, °)

*D*—H⋯*A*	*D*—H	H⋯*A*	*D*⋯*A*	*D*—H⋯*A*
O4—H4⋯O7^i^	0.82	1.64	2.443 (2)	168
C2—H2⋯O6^ii^	0.93	2.51	3.326 (2)	147
C4—H4*A*⋯O5^iii^	0.93	2.54	3.341 (2)	145
C7—H7⋯O5^iii^	0.93	2.51	3.304 (2)	143
C9—H9⋯O8^iv^	0.93	2.36	3.097 (2)	136
C12—H12⋯O1^v^	0.93	2.59	3.194 (2)	123
C17—H17⋯O7^vi^	0.93	2.42	3.148 (2)	136

Symmetry codes: (i) 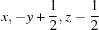; (ii) 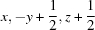; (iii) 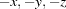; (iv) 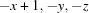; (v) 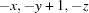; (vi) 
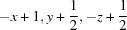
.

**Fig. 1.**
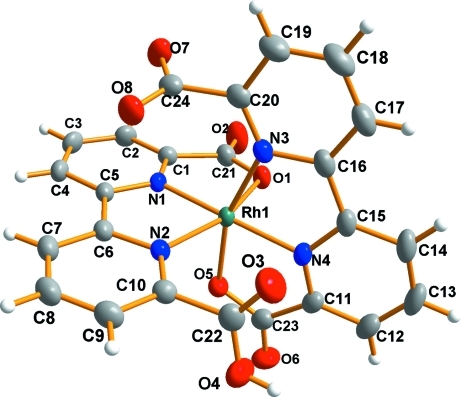
The structure of the title compound with 50% probability displacement ellipsoids.

**Fig. 2.**
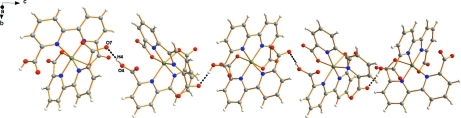
The one-dimensional chain structure of the title compound via hydrogen bonds along the c-axis.

## supplementary crystallographic information

### Comment

Thanks to diverse coordination modes and aromatic cores, many multidentate ligands containing N- or O-donors, such as pyridine-2,6-dicarboxylic acid, 2,2'-dipyridine-4,4'-dicarboxylic acid and 2,2'-dipyridine-5,5'-dicarboxylic acid, have been widely used in metal–organic coordination chemistry (Robin & Fromm, 2006; Wang *et al.*, 2009). However, the study of complexes with the title ligand (*H_2_L* = 2,2'-bipyridine-6,6'-dicarboxylic acid) is still rare. Some X-ray crystal structures constructed from the title ligand and metal ions, such as [Ln_2_*L*_3_(H_2_O)_3_].xH_2_O (*x* = 1, Ln = Eu, Tb; *x* = 0, Ln = Gd) (Bünzli *et al.*, 2000), [Ln_3_*L*_4_(HL)(H_2_O)_2_].12H_2_O (Ln = Ce, Nd, Pr) (Wang *et al.*, 2010), [Ln_2_*L*_3_(H_2_O)_3_].3H_2_O (Ln = Er, Tm) (Wang *et al.*, 2010), [Ni*L*_2_].4H_2_O (Wang *et al.*, 2009) and [CdL]_n_.2nH_2_O (Knight *et al.*, 2006), have been investigated previously. Here we isolated a new compound constructed from the title ligand and rhodium(III) under hydrothermal conditions. A careful literature survey showed that it is the first compound constructed from rhodium(III) and the title ligand.

The structure of the title compound (Fig. 1) shows that the six-coordinated Rh^III^ atom is surrounded by four N atoms and two O atoms from the two chelated ligands to form a distorted octahedral geometry. The Rh—N bond lengths are in the range of 1.9529 (13)–2.0808 (14) Å and the Rh—O bond lengths are 2.0226 (12) and 2.0316 (13) Å (Table 1), which are comparable to other distances reported in Rh^III^ complexes (Burke *et al.*, 2004; Chapman *et al.*, 2002). The coordinated bipyridine fragments are nearly coplanar [see torsion angles of 2.1 (2) and 1.8 (2)° in Table 1].

Face-to-face π-stacking interactions between inversion-related pyridine rings with *Cg*1···*Cg*2^ii^ distance of 3.581 (1) Å (the perpendicular distance between the rings is 3.3980 (7) Å) are observed in the crystal structure: *Cg*1 and *Cg*2 are the centroids of the pyridine rings (N1,C1–C5) and (N2,C6–C10), respectively (symmetry code: ii = -*x*, -*y*, -*z*). A similar situation of face-to-face π-stacking interactions was observed in our early work (Wang *et al.*, 2009).

The one-dimensional chain structure of the title compound *via* hydrogen bonds is illustrated in Fig. 2. The hydrogen-bond donor O4 is connected to the acceptor O7 from the adjacent molecule to form one-dimensional infinite chains along the *c*-axis (O4—H4···O7^i^ = 1.64 Å, i = *x*, -*y* + 1/2, *z* - 1/2, Table 2). Several intermolecular C—H···O interactions contribute to stabilize the crystal structure.

### Experimental

The title compound was obtained by the reaction of the mixture of RhCl_3_ and 2,2'-dipyridine-6,6'-dicarboxylic acid in a molar ratio of 1:3 and 10 ml of water under hydrothermal conditions (at 433 K for 3 d and cooled to room temperature with a 3°C h^-1^ rate). The orange block crystals were washed with water (yield: 40%).

### Refinement

The H atoms were placed in geometrically idealized positions (C—H = 0.95 Å and O—H = 0.82–0.84 Å) with *U*_iso_(H) = 1.2*U*_eq_(C) and *U*_iso_(H) = 1.5*U*_eq_(O).

### Crystal data

**Table:**

[Rh(C_12_H_6_N_2_O_4_)(C_12_H_7_N_2_O_4_)]	*Z* = 4
*M**_r_* = 588.29	*F*(000) = 1176
Monoclinic, *P*2_1_/*c*	*D*_x_ = 1.841 Mg m^−^^3^
Hall symbol: -P 2ybc	Mo *K*α radiation, λ = 0.71073 Å
*a* = 9.3308 (4) Å	µ = 0.87 mm^−^^1^
*b* = 13.6186 (6) Å	*T* = 296 K
*c* = 16.9974 (8) Å	Block, orange
β = 100.696 (1)°	0.2 × 0.2 × 0.2 mm
*V* = 2122.37 (16) Å^3^	

### Data collection

**Table:**

Bruker APEXII CCD area-detector diffractometer	4714 reflections with *I* > 2σ(*I*)
Radiation source: fine-focus sealed tube	*R*_int_ = 0.017
Graphite monochromator	θ_max_ = 28.3°, θ_min_ = 1.9°
φ and ω scans	*h* = −10→12
15424 measured reflections	*k* = −17→18
5269 independent reflections	*l* = −22→20

### Refinement

**Table:**

Refinement on *F*^2^	Primary atom site location: structure-invariant direct methods
Least-squares matrix: full	Secondary atom site location: difference Fourier map
*R*[*F*^2^ > 2σ(*F*^2^)] = 0.023	Hydrogen site location: inferred from neighbouring sites
*wR*(*F*^2^) = 0.061	H-atom parameters constrained
*S* = 1.03	*w* = 1/[σ^2^(*F*_o_^2^) + (0.0317*P*)^2^ + 1.1072*P*] where *P* = (*F*_o_^2^ + 2*F*_c_^2^)/3
5269 reflections	(Δ/σ)_max_ < 0.001
335 parameters	Δρ_max_ = 0.42 e Å^−^^3^
0 restraints	Δρ_min_ = −0.58 e Å^−^^3^

### Special details

**Table:**

Geometry. All e.s.d.'s (except the e.s.d. in the dihedral angle between two l.s. planes) are estimated using the full covariance matrix. The cell e.s.d.'s are taken into account individually in the estimation of e.s.d.'s in distances, angles and torsion angles; correlations between e.s.d.'s in cell parameters are only used when they are defined by crystal symmetry. An approximate (isotropic) treatment of cell e.s.d.'s is used for estimating e.s.d.'s involving l.s. planes.
Refinement. Refinement of *F*^2^ against ALL reflections. The weighted *R*-factor *wR* and goodness of fit *S* are based on *F*^2^, conventional *R*-factors *R* are based on *F*, with *F* set to zero for negative *F*^2^. The threshold expression of *F*^2^ > σ(*F*^2^) is used only for calculating *R*-factors(gt) *etc*. and is not relevant to the choice of reflections for refinement. *R*-factors based on *F*^2^ are statistically about twice as large as those based on *F*, and *R*-factors based on ALL data will be even larger.

### Fractional atomic coordinates and isotropic or equivalent isotropic displacement parameters (Å^2^)

**Table:**

	*x*	*y*	*z*	*U*_iso_*/*U*_eq_	
Rh1	0.159112 (14)	0.237745 (9)	0.067212 (7)	0.02121 (5)	
N1	0.08891 (15)	0.12682 (10)	0.12226 (8)	0.0225 (3)	
N2	0.24130 (15)	0.12072 (10)	0.01055 (8)	0.0237 (3)	
O5	−0.02277 (15)	0.23972 (9)	−0.01957 (8)	0.0298 (3)	
N4	0.19640 (17)	0.35997 (10)	0.01389 (9)	0.0265 (3)	
C6	0.20550 (18)	0.03125 (12)	0.03847 (10)	0.0250 (3)	
C5	0.11551 (18)	0.03481 (12)	0.10163 (10)	0.0241 (3)	
O1	0.05205 (14)	0.31499 (9)	0.13993 (8)	0.0287 (3)	
N3	0.35387 (16)	0.27865 (11)	0.13906 (9)	0.0261 (3)	
O4	0.26900 (19)	0.24099 (12)	−0.14737 (10)	0.0473 (4)	
H4	0.3000	0.2889	−0.1681	0.071*	
C4	0.0559 (2)	−0.04235 (13)	0.13826 (11)	0.0298 (4)	
H4A	0.0739	−0.1071	0.1258	0.036*	
C21	−0.0203 (2)	0.25889 (13)	0.18174 (12)	0.0297 (4)	
C11	0.0918 (2)	0.38968 (13)	−0.04605 (11)	0.0299 (4)	
O7	0.32750 (17)	0.11501 (10)	0.27890 (8)	0.0415 (3)	
C20	0.4329 (2)	0.23263 (13)	0.20224 (11)	0.0297 (4)	
O8	0.42615 (17)	0.06416 (10)	0.17725 (9)	0.0409 (3)	
C24	0.3925 (2)	0.12843 (13)	0.21996 (11)	0.0299 (4)	
O3	0.4556 (2)	0.26903 (12)	−0.04658 (11)	0.0534 (4)	
O6	−0.13732 (17)	0.33511 (12)	−0.11836 (9)	0.0457 (4)	
C1	0.00247 (19)	0.14967 (13)	0.17412 (10)	0.0257 (3)	
C15	0.3070 (2)	0.41706 (13)	0.04922 (11)	0.0305 (4)	
C3	−0.0313 (2)	−0.02061 (14)	0.19393 (11)	0.0330 (4)	
H3	−0.0709	−0.0715	0.2195	0.040*	
C7	0.2492 (2)	−0.05583 (13)	0.00854 (11)	0.0314 (4)	
H7	0.2245	−0.1157	0.0287	0.038*	
C9	0.3635 (2)	0.03691 (15)	−0.08131 (12)	0.0344 (4)	
H9	0.4161	0.0403	−0.1227	0.041*	
C16	0.3976 (2)	0.37045 (13)	0.11932 (12)	0.0308 (4)	
C23	−0.0336 (2)	0.31820 (14)	−0.06556 (11)	0.0317 (4)	
C8	0.3306 (2)	−0.05271 (15)	−0.05210 (12)	0.0349 (4)	
H8	0.3623	−0.1104	−0.0726	0.042*	
O2	−0.10279 (18)	0.29013 (11)	0.22277 (11)	0.0498 (4)	
C18	0.5979 (2)	0.36721 (17)	0.22862 (14)	0.0457 (5)	
H18	0.6798	0.3969	0.2588	0.055*	
C2	−0.0601 (2)	0.07623 (14)	0.21197 (11)	0.0320 (4)	
H2	−0.1199	0.0909	0.2485	0.038*	
C13	0.2164 (3)	0.54139 (15)	−0.04621 (14)	0.0434 (5)	
H13	0.2255	0.6030	−0.0684	0.052*	
C10	0.31789 (19)	0.12252 (13)	−0.04881 (11)	0.0277 (3)	
C14	0.3194 (2)	0.51125 (14)	0.01885 (14)	0.0399 (5)	
H14	0.3949	0.5527	0.0417	0.048*	
C12	0.1004 (2)	0.48196 (14)	−0.07888 (12)	0.0386 (4)	
H12	0.0303	0.5034	−0.1216	0.046*	
C19	0.5562 (2)	0.27527 (16)	0.24857 (14)	0.0419 (5)	
H19	0.6092	0.2420	0.2922	0.050*	
C22	0.3551 (2)	0.22076 (15)	−0.08135 (11)	0.0321 (4)	
C17	0.5179 (2)	0.41533 (15)	0.16363 (13)	0.0400 (5)	
H17	0.5452	0.4778	0.1499	0.048*	

### Atomic displacement parameters (Å^2^)

**Table:**

	*U*^11^	*U*^22^	*U*^33^	*U*^12^	*U*^13^	*U*^23^
Rh1	0.02553 (8)	0.01557 (7)	0.02292 (7)	−0.00143 (4)	0.00549 (5)	−0.00021 (4)
N1	0.0264 (7)	0.0176 (6)	0.0235 (7)	−0.0014 (5)	0.0043 (5)	0.0010 (5)
N2	0.0262 (7)	0.0203 (6)	0.0246 (7)	−0.0007 (5)	0.0046 (6)	−0.0018 (5)
O5	0.0323 (7)	0.0257 (6)	0.0296 (7)	−0.0020 (5)	0.0010 (5)	0.0006 (5)
N4	0.0340 (8)	0.0190 (7)	0.0284 (7)	−0.0008 (6)	0.0108 (6)	0.0010 (5)
C6	0.0266 (8)	0.0215 (8)	0.0258 (8)	−0.0009 (6)	0.0021 (7)	−0.0012 (6)
C5	0.0275 (8)	0.0195 (7)	0.0240 (8)	−0.0005 (6)	0.0014 (6)	−0.0003 (6)
O1	0.0349 (7)	0.0197 (6)	0.0336 (7)	0.0008 (5)	0.0119 (5)	−0.0026 (5)
N3	0.0277 (7)	0.0221 (7)	0.0289 (7)	−0.0024 (6)	0.0063 (6)	−0.0041 (6)
O4	0.0565 (10)	0.0437 (9)	0.0410 (9)	−0.0150 (7)	0.0075 (7)	0.0115 (6)
C4	0.0366 (10)	0.0196 (8)	0.0317 (9)	−0.0019 (7)	0.0019 (7)	0.0027 (7)
C21	0.0312 (9)	0.0253 (8)	0.0332 (9)	−0.0002 (7)	0.0079 (7)	−0.0035 (7)
C11	0.0402 (10)	0.0248 (8)	0.0268 (9)	0.0052 (7)	0.0114 (7)	0.0022 (7)
O7	0.0612 (9)	0.0309 (7)	0.0357 (7)	0.0079 (7)	0.0174 (7)	0.0000 (6)
C20	0.0286 (9)	0.0299 (9)	0.0299 (9)	0.0033 (7)	0.0038 (7)	−0.0067 (7)
O8	0.0523 (9)	0.0313 (7)	0.0405 (8)	0.0085 (6)	0.0122 (7)	−0.0078 (6)
C24	0.0309 (9)	0.0282 (9)	0.0279 (9)	0.0074 (7)	−0.0017 (7)	−0.0018 (7)
O3	0.0542 (10)	0.0489 (10)	0.0556 (10)	−0.0211 (8)	0.0065 (8)	−0.0042 (8)
O6	0.0470 (9)	0.0529 (9)	0.0332 (7)	0.0040 (7)	−0.0036 (6)	0.0070 (7)
C1	0.0287 (8)	0.0245 (8)	0.0241 (8)	−0.0007 (6)	0.0054 (7)	−0.0010 (6)
C15	0.0354 (10)	0.0216 (8)	0.0375 (10)	−0.0036 (7)	0.0147 (8)	−0.0020 (7)
C3	0.0392 (10)	0.0278 (9)	0.0321 (9)	−0.0053 (7)	0.0072 (8)	0.0079 (7)
C7	0.0356 (10)	0.0226 (8)	0.0348 (10)	0.0005 (7)	0.0036 (8)	−0.0027 (7)
C9	0.0336 (10)	0.0385 (11)	0.0330 (10)	0.0039 (8)	0.0109 (8)	−0.0068 (8)
C16	0.0324 (9)	0.0247 (8)	0.0374 (10)	−0.0050 (7)	0.0116 (8)	−0.0067 (7)
C23	0.0390 (10)	0.0301 (9)	0.0266 (9)	0.0047 (8)	0.0080 (8)	−0.0009 (7)
C8	0.0359 (10)	0.0306 (10)	0.0379 (10)	0.0073 (8)	0.0057 (8)	−0.0094 (8)
O2	0.0586 (10)	0.0346 (8)	0.0667 (11)	0.0002 (7)	0.0390 (9)	−0.0092 (7)
C18	0.0322 (10)	0.0469 (13)	0.0547 (13)	−0.0081 (9)	−0.0003 (9)	−0.0187 (10)
C2	0.0367 (10)	0.0336 (9)	0.0275 (9)	−0.0027 (8)	0.0109 (7)	0.0024 (7)
C13	0.0599 (14)	0.0240 (9)	0.0528 (13)	0.0031 (9)	0.0273 (11)	0.0111 (8)
C10	0.0272 (8)	0.0289 (9)	0.0272 (8)	−0.0008 (7)	0.0054 (7)	−0.0024 (7)
C14	0.0459 (12)	0.0233 (9)	0.0554 (13)	−0.0066 (8)	0.0224 (10)	0.0000 (8)
C12	0.0537 (12)	0.0296 (10)	0.0354 (10)	0.0080 (9)	0.0159 (9)	0.0084 (8)
C19	0.0343 (10)	0.0438 (12)	0.0427 (12)	0.0021 (9)	−0.0055 (9)	−0.0096 (9)
C22	0.0350 (10)	0.0329 (10)	0.0328 (10)	−0.0022 (7)	0.0179 (8)	−0.0034 (7)
C17	0.0366 (11)	0.0323 (10)	0.0518 (12)	−0.0106 (8)	0.0101 (9)	−0.0113 (9)

### Geometric parameters (Å, º)

**Table:**

Rh1—N1	1.9529 (13)	C20—C24	1.513 (3)
Rh1—N4	1.9571 (14)	O8—C24	1.215 (2)
Rh1—O1	2.0226 (12)	O3—C22	1.206 (3)
Rh1—O5	2.0316 (13)	O6—C23	1.213 (2)
Rh1—N3	2.0689 (15)	C1—C2	1.377 (2)
Rh1—N2	2.0808 (14)	C15—C14	1.396 (3)
N1—C5	1.337 (2)	C15—C16	1.471 (3)
N1—C1	1.337 (2)	C3—C2	1.391 (3)
N2—C10	1.341 (2)	C3—H3	0.9300
N2—C6	1.371 (2)	C7—C8	1.390 (3)
O5—C23	1.317 (2)	C7—H7	0.9300
N4—C11	1.336 (2)	C9—C8	1.374 (3)
N4—C15	1.342 (2)	C9—C10	1.390 (3)
C6—C7	1.382 (2)	C9—H9	0.9300
C6—C5	1.481 (2)	C16—C17	1.374 (3)
C5—C4	1.389 (2)	C8—H8	0.9300
O1—C21	1.313 (2)	C18—C19	1.372 (3)
N3—C20	1.340 (2)	C18—C17	1.379 (3)
N3—C16	1.376 (2)	C18—H18	0.9300
O4—C22	1.283 (3)	C2—H2	0.9300
O4—H4	0.8200	C13—C12	1.382 (3)
C4—C3	1.389 (3)	C13—C14	1.386 (3)
C4—H4A	0.9300	C13—H13	0.9300
C21—O2	1.208 (2)	C10—C22	1.513 (3)
C21—C1	1.512 (2)	C14—H14	0.9300
C11—C12	1.383 (3)	C12—H12	0.9300
C11—C23	1.511 (3)	C19—H19	0.9300
O7—C24	1.277 (2)	C17—H17	0.9300
C20—C19	1.394 (3)		
			
N1—Rh1—N4	169.74 (6)	N1—C1—C2	119.94 (16)
N1—Rh1—O1	82.06 (5)	N1—C1—C21	113.40 (15)
N4—Rh1—O1	89.49 (5)	C2—C1—C21	126.61 (16)
N1—Rh1—O5	92.80 (5)	N4—C15—C14	118.48 (18)
N4—Rh1—O5	81.31 (6)	N4—C15—C16	113.04 (15)
O1—Rh1—O5	89.77 (5)	C14—C15—C16	128.44 (18)
N1—Rh1—N3	105.16 (6)	C4—C3—C2	120.91 (17)
N4—Rh1—N3	80.34 (6)	C4—C3—H3	119.5
O1—Rh1—N3	88.77 (5)	C2—C3—H3	119.5
O5—Rh1—N3	161.60 (5)	C6—C7—C8	119.09 (18)
N1—Rh1—N2	79.31 (6)	C6—C7—H7	120.5
N4—Rh1—N2	108.94 (6)	C8—C7—H7	120.5
O1—Rh1—N2	161.35 (5)	C8—C9—C10	119.73 (18)
O5—Rh1—N2	90.23 (5)	C8—C9—H9	120.1
N3—Rh1—N2	96.93 (6)	C10—C9—H9	120.1
C5—N1—C1	123.71 (15)	C17—C16—N3	121.32 (19)
C5—N1—Rh1	120.29 (12)	C17—C16—C15	122.71 (17)
C1—N1—Rh1	115.65 (11)	N3—C16—C15	115.96 (16)
C10—N2—C6	118.36 (15)	O6—C23—O5	123.79 (19)
C10—N2—Rh1	128.91 (12)	O6—C23—C11	121.25 (18)
C6—N2—Rh1	112.71 (11)	O5—C23—C11	114.92 (16)
C23—O5—Rh1	113.81 (12)	C9—C8—C7	119.00 (17)
C11—N4—C15	123.93 (15)	C9—C8—H8	120.5
C11—N4—Rh1	116.31 (12)	C7—C8—H8	120.5
C15—N4—Rh1	118.27 (12)	C19—C18—C17	119.67 (19)
N2—C6—C7	121.85 (16)	C19—C18—H18	120.2
N2—C6—C5	115.40 (14)	C17—C18—H18	120.2
C7—C6—C5	122.74 (15)	C1—C2—C3	118.00 (17)
N1—C5—C4	118.83 (16)	C1—C2—H2	121.0
N1—C5—C6	112.24 (14)	C3—C2—H2	121.0
C4—C5—C6	128.90 (15)	C12—C13—C14	121.48 (18)
C21—O1—Rh1	112.97 (10)	C12—C13—H13	119.3
C20—N3—C16	118.43 (16)	C14—C13—H13	119.3
C20—N3—Rh1	129.77 (12)	N2—C10—C9	121.95 (17)
C16—N3—Rh1	111.63 (12)	N2—C10—C22	118.83 (16)
C22—O4—H4	109.5	C9—C10—C22	119.23 (16)
C5—C4—C3	118.54 (16)	C13—C14—C15	118.33 (19)
C5—C4—H4A	120.7	C13—C14—H14	120.8
C3—C4—H4A	120.7	C15—C14—H14	120.8
O2—C21—O1	123.68 (17)	C13—C12—C11	118.02 (19)
O2—C21—C1	120.73 (17)	C13—C12—H12	121.0
O1—C21—C1	115.58 (15)	C11—C12—H12	121.0
N4—C11—C12	119.57 (18)	C18—C19—C20	118.9 (2)
N4—C11—C23	113.49 (15)	C18—C19—H19	120.6
C12—C11—C23	126.69 (18)	C20—C19—H19	120.6
N3—C20—C19	122.11 (18)	O3—C22—O4	128.0 (2)
N3—C20—C24	118.53 (16)	O3—C22—C10	120.88 (19)
C19—C20—C24	119.22 (18)	O4—C22—C10	111.14 (16)
O8—C24—O7	125.35 (18)	C16—C17—C18	119.59 (19)
O8—C24—C20	117.09 (17)	C16—C17—H17	120.2
O7—C24—C20	117.56 (16)	C18—C17—H17	120.2
			
N4—Rh1—N1—C5	142.4 (3)	Rh1—N4—C11—C12	170.67 (14)
O1—Rh1—N1—C5	177.26 (13)	C15—N4—C11—C23	−169.87 (16)
O5—Rh1—N1—C5	87.88 (13)	Rh1—N4—C11—C23	−4.10 (19)
N3—Rh1—N1—C5	−96.19 (13)	C16—N3—C20—C19	0.7 (3)
N2—Rh1—N1—C5	−1.83 (12)	Rh1—N3—C20—C19	−174.12 (15)
N4—Rh1—N1—C1	−31.0 (4)	C16—N3—C20—C24	−174.99 (16)
O1—Rh1—N1—C1	3.86 (12)	Rh1—N3—C20—C24	10.2 (2)
O5—Rh1—N1—C1	−85.52 (12)	N3—C20—C24—O8	75.6 (2)
N3—Rh1—N1—C1	90.41 (13)	C19—C20—C24—O8	−100.2 (2)
N2—Rh1—N1—C1	−175.23 (13)	N3—C20—C24—O7	−105.1 (2)
N1—Rh1—N2—C10	178.45 (16)	C19—C20—C24—O7	79.0 (2)
N4—Rh1—N2—C10	4.76 (16)	C5—N1—C1—C2	2.6 (3)
O1—Rh1—N2—C10	175.62 (15)	Rh1—N1—C1—C2	175.79 (13)
O5—Rh1—N2—C10	85.64 (15)	C5—N1—C1—C21	−174.92 (15)
N3—Rh1—N2—C10	−77.37 (15)	Rh1—N1—C1—C21	−1.77 (19)
N1—Rh1—N2—C6	0.49 (11)	O2—C21—C1—N1	175.54 (19)
N4—Rh1—N2—C6	−173.19 (11)	O1—C21—C1—N1	−3.0 (2)
O1—Rh1—N2—C6	−2.3 (2)	O2—C21—C1—C2	−1.8 (3)
O5—Rh1—N2—C6	−92.31 (12)	O1—C21—C1—C2	179.66 (17)
N3—Rh1—N2—C6	104.68 (12)	C11—N4—C15—C14	−4.0 (3)
N1—Rh1—O5—C23	168.90 (13)	Rh1—N4—C15—C14	−169.55 (14)
N4—Rh1—O5—C23	−2.66 (12)	C11—N4—C15—C16	173.52 (16)
O1—Rh1—O5—C23	86.86 (12)	Rh1—N4—C15—C16	8.0 (2)
N3—Rh1—O5—C23	1.4 (2)	C5—C4—C3—C2	0.9 (3)
N2—Rh1—O5—C23	−111.79 (13)	N2—C6—C7—C8	−0.6 (3)
N1—Rh1—N4—C11	−51.6 (4)	C5—C6—C7—C8	178.45 (16)
O1—Rh1—N4—C11	−86.07 (13)	C20—N3—C16—C17	−1.0 (3)
O5—Rh1—N4—C11	3.78 (12)	Rh1—N3—C16—C17	174.67 (15)
N3—Rh1—N4—C11	−174.92 (13)	C20—N3—C16—C15	179.72 (16)
N2—Rh1—N4—C11	91.02 (13)	Rh1—N3—C16—C15	−4.57 (19)
N1—Rh1—N4—C15	115.0 (3)	N4—C15—C16—C17	178.97 (17)
O1—Rh1—N4—C15	80.54 (13)	C14—C15—C16—C17	−3.8 (3)
O5—Rh1—N4—C15	170.39 (14)	N4—C15—C16—N3	−1.8 (2)
N3—Rh1—N4—C15	−8.30 (13)	C14—C15—C16—N3	175.46 (18)
N2—Rh1—N4—C15	−102.37 (13)	Rh1—O5—C23—O6	−176.56 (16)
C10—N2—C6—C7	1.6 (2)	Rh1—O5—C23—C11	1.25 (19)
Rh1—N2—C6—C7	179.84 (14)	N4—C11—C23—O6	179.66 (17)
C10—N2—C6—C5	−177.49 (15)	C12—C11—C23—O6	5.3 (3)
Rh1—N2—C6—C5	0.70 (18)	N4—C11—C23—O5	1.8 (2)
C1—N1—C5—C4	−2.9 (3)	C12—C11—C23—O5	−172.54 (18)
Rh1—N1—C5—C4	−175.79 (12)	C10—C9—C8—C7	1.4 (3)
C1—N1—C5—C6	175.50 (15)	C6—C7—C8—C9	−0.9 (3)
Rh1—N1—C5—C6	2.7 (2)	N1—C1—C2—C3	−0.5 (3)
N2—C6—C5—N1	−2.1 (2)	C21—C1—C2—C3	176.71 (18)
C7—C6—C5—N1	178.79 (16)	C4—C3—C2—C1	−1.2 (3)
N2—C6—C5—C4	176.16 (17)	C6—N2—C10—C9	−1.2 (3)
C7—C6—C5—C4	−3.0 (3)	Rh1—N2—C10—C9	−179.03 (13)
N1—Rh1—O1—C21	−5.47 (13)	C6—N2—C10—C22	178.66 (15)
N4—Rh1—O1—C21	168.69 (13)	Rh1—N2—C10—C22	0.8 (2)
O5—Rh1—O1—C21	87.39 (13)	C8—C9—C10—N2	−0.3 (3)
N3—Rh1—O1—C21	−110.96 (13)	C8—C9—C10—C22	179.85 (18)
N2—Rh1—O1—C21	−2.7 (2)	C12—C13—C14—C15	2.5 (3)
N1—Rh1—N3—C20	10.65 (17)	N4—C15—C14—C13	0.3 (3)
N4—Rh1—N3—C20	−178.22 (16)	C16—C15—C14—C13	−176.87 (19)
O1—Rh1—N3—C20	92.09 (16)	C14—C13—C12—C11	−1.7 (3)
O5—Rh1—N3—C20	177.68 (16)	N4—C11—C12—C13	−1.9 (3)
N2—Rh1—N3—C20	−70.10 (16)	C23—C11—C12—C13	172.14 (18)
N1—Rh1—N3—C16	−164.44 (12)	C17—C18—C19—C20	0.0 (3)
N4—Rh1—N3—C16	6.69 (12)	N3—C20—C19—C18	−0.2 (3)
O1—Rh1—N3—C16	−83.00 (12)	C24—C20—C19—C18	175.48 (19)
O5—Rh1—N3—C16	2.6 (2)	N2—C10—C22—O3	79.9 (2)
N2—Rh1—N3—C16	114.81 (12)	C9—C10—C22—O3	−100.3 (2)
N1—C5—C4—C3	1.1 (3)	N2—C10—C22—O4	−100.8 (2)
C6—C5—C4—C3	−177.02 (17)	C9—C10—C22—O4	79.0 (2)
Rh1—O1—C21—O2	−172.48 (18)	N3—C16—C17—C18	0.9 (3)
Rh1—O1—C21—C1	6.0 (2)	C15—C16—C17—C18	−179.95 (19)
C15—N4—C11—C12	4.9 (3)	C19—C18—C17—C16	−0.3 (3)

### Hydrogen-bond geometry (Å, º)

**Table:**

*D*—H···*A*	*D*—H	H···*A*	*D*···*A*	*D*—H···*A*
O4—H4···O7^i^	0.82	1.64	2.443 (2)	168
C2—H2···O6^ii^	0.93	2.51	3.326 (2)	147
C4—H4*A*···O5^iii^	0.93	2.54	3.341 (2)	145
C7—H7···O5^iii^	0.93	2.51	3.304 (2)	143
C9—H9···O8^iv^	0.93	2.36	3.097 (2)	136
C12—H12···O1^v^	0.93	2.59	3.194 (2)	123
C17—H17···O7^vi^	0.93	2.42	3.148 (2)	136

Symmetry codes: (i) *x*, −*y*+1/2, *z*−1/2; (ii) *x*, −*y*+1/2, *z*+1/2; (iii) −*x*, −*y*, −*z*; (iv) −*x*+1, −*y*, −*z*; (v) −*x*, −*y*+1, −*z*; (vi) −*x*+1, *y*+1/2, −*z*+1/2.
